# Prevalence of Contraindications to Nirmatrelvir-Ritonavir Among Hospitalized Patients With COVID-19 at Risk for Progression to Severe Disease

**DOI:** 10.1001/jamanetworkopen.2022.42140

**Published:** 2022-11-15

**Authors:** Nicolas Hoertel, David R. Boulware, Marina Sánchez-Rico, Anita Burgun, Frédéric Limosin

**Affiliations:** 1 Université Paris Cité, Paris, France; 2 INSERM U1266, Institut de Psychiatrie et Neuroscience de Paris, Paris, France; 3 Assistance Publique–Hôpitaux de Paris, DMU Psychiatrie et Addictologie, Service de Psychiatrie et Addictologie, Hôpital Corentin-Celton, Issy-les-Moulineaux, France; 4 Division of Infectious Diseases and International Medicine, Department of Medicine, University of Minnesota, Minneapolis; 5 INSERM, UMR S1138, Cordeliers Research Center, Université de Paris, Paris, France

## Abstract

This cohort study examines the prevalence of contraindications to nirmatrelvir-ritonavir in patients hospitalized with COVID-19.

## Introduction

The availability of outpatient treatment options for COVID-19, such as nirmatrelvir-ritonavir, remdesivir, molnupiravir, and monoclonal antibodies, raises hopes for reducing COVID-19–related morbidity and mortality. Among these options, nirmatrelvir-ritonavir (Paxlovid)^1^ may be preferred for most high-risk patients because of the convenience of oral dosing and its high efficacy vs placebo in reducing hospitalization or death, as reported in a phase 2/3 trial.^1^

However, there are circumstances in which nirmatrelvir-ritonavir should not be used, due to the effect of ritonavir. Ritonavir may elevate the concentrations of drugs highly dependent on hepatic cytochrome P-450 3A (CYP3A) metabolism, potentially resulting in serious reactions. Conversely, coadministration with potent CYP3A inducers can significantly reduce nirmatrelvir concentrations, potentially leading to the loss of virologic response. Furthermore, patients with severe kidney impairment and severe liver impairment were excluded from the clinical trials.^1^ These medical contraindications may be prevalent in patients with COVID-19 who are at high risk for progression to severe disease, as suggested by a recent study.^2,3^

We examined the prevalence of contraindications to nirmatrelvir-ritonavir in patients hospitalized with COVID-19. We hypothesized that the rate would be high in these patients.

## Methods

In this cohort study, we applied individual medical contraindications listed by the US Food and Drug Administration for nirmatrelvir-ritonavir to a large sample of patients hospitalized with COVID-19, ascertained by a positive reverse transcription–polymerase chain reaction test, in 36 greater Paris University hospitals from January 24, 2020, to November 30, 2021.^4,5,6^ No patients received nirmatrelvir-ritonavir. We examined the proportion of patients with contraindications to nirmatrelvir-ritonavir in this sample and in those who died within 28 days of hospital admission, who thereby would have needed therapy other than nirmatrelvir-ritonavir in the ambulatory setting. We also stratified the analysis by sex, age (≤65 y vs >65 y), and comorbidity based on *International Statistical Classification of Diseases and Related Health Problems, Tenth Revision *(*ICD-10*) main chapters. Medical contraindications of nirmatrelvir-ritonavir and criteria used to approximate them are detailed in Table 1. Analyses were performed using R software version 3.6.3. This study received approval from the institutional review board of the Assistance Publique–Hôpitaux de Paris clinical data warehouse. Patient consent was not applicable because this study did not contain factors necessitating it. The study was performed in accordance with STROBE reporting guidelines.

**Table 1.** Prevalence of Possible Medical Contraindications to Nirmatrelvir-Ritonavir in a Sample of Patients Hospitalized With COVID-19

Medical contraindications listed by the US FDA^a^	Patients, No. (%)									
	Full sample of hospitalized patients					Subsample of patients who died within 28 d of hospital admission				
	Total (N = 62 525)	Women (n = 31 561)	Men (n = 30 964)	Age ≤65 y (n = 42 461)	Age >65 y (n = 20 064)	Total (n = 4861)	Women (n = 1882)	Men (n = 2979)	Age ≤65 y (n = 768)	Age >65 y (n = 4093)
Use of medications dependent on CYP3A for clearance^b^										
Any	3233 (5.15)	1218 (3.86)	2015 (6.51)	548 (1.29)	2685 (13.4)	1340 (27.6)	558 (29.6)	782 (26.3)	85 (11.1)	1255 (30.7)
α1-Adrenoreceptor antagonist: alfuzosin	789 (1.26)	72 (0.23)	717 (2.32)	202 (0.48)	587 (2.93)	127 (2.61)	8 (0.43)	119 (3.99)	9 (1.17)	118 (2.88)
Antiarrhythmic: amiodarone, dronedarone, flecainide, propafenone, quinidine	734 (1.17)	319 (1.01)	415 (1.34)	84 (0.20)	650 (3.24)	198 (4.07)	91 (4.84)	107 (3.59)	12 (1.56)	186 (4.54)
HMG-CoA reductase inhibitors: lovastatin, simvastatin	389 (0.62)	155 (0.49)	234 (0.76)	73 (0.17)	316 (1.57)	67 (1.38)	19 (1.01)	48 (1.61)	2 (0.26)	65 (1.59)
Other medications^c^	206 (0.33)	94 (0.30)	112 (0.36)	65 (0.15)	141 (0.70)	23 (0.47)	8 (0.43)	15 (0.50)	3 (0.39)	20 (0.49)
Use of medications that induce CYP3A^b^^,^^d^	145 (0.23)	68 (0.22)	77 (0.25)	55 (0.13)	90 (0.45)	18 (0.37)	7 (0.37)	11 (0.37)	5 (0.65)	13 (0.32)
Severe hepatic impairment^e^^,^^f^	832 (1.33)	303 (0.96)	529 (1.71)	334 (0.79)	498 (2.48)	282 (5.80)	97 (5.15)	183 (6.14)	92 (12.0)	188 (4.59)
Severe kidney impairment^f^^,^^g^	3958 (6.33)	1453 (4.60)	2505 (8.09)	1259 (2.97)	2699 (13.5)	1243 (25.6)	398 (21.1)	845 (28.4)	266 (34.6)	977 (23.9)
Age <12 y	1684 (2.69)	729 (2.31)	955 (3.08)	1684 (3.97)	Not applicable	7 (0.14)	3 (0.16)	4 (0.13)	7 (0.91)	Not applicable
At least 1 medical contraindication to nirmatrelvir-ritonavir^h^	9136 (14.6)	3577 (11.3)	5568 (18.0)	3738 (8.80)	5398 (26.9)	2463 (50.7)	922 (49.0)	1538 (51.6)	381 (49.6)	2082 (50.9)

Abbreviations: CYP3A, cytochrome P 3A; FDA, Food and Drug Administration; HMG-CoA, β-Hydroxy β-methylglutaryl-CoA.

^a^ Data on the medical contraindications of weight less than 40 kg, age younger than 18 years, and history of clinically significant hypersensitive reactions to nirmatrelvir or ritonavir were not available.

^b^ Based on prescription data at hospital admission.

^c^ Medications with less than 0.5% overall use in the full sample of hospitalized patients with COVID-19 were grouped into a category other medications; they included analgesics (pethidine, piroxicam, propoxyphene), antianginal (ranolazine), anti-gout (colchicine), antipsychotics (lurasidone, pimozide, clozapine), ergot derivatives (dihydroergotamine, ergotamine, methylergonovine), PDE5 inhibitors (sildenafil when used for pulmonary arterial hypertension), and sedative/hypnotics (triazolam, oral midazolam).

^d^ This category includes anticancer drugs (apalutamide), anticonvulsants (carbamazepine, phenobarbital, phenytoin), and antimycobacterials (rifampin, herbal products, such as St John’s Wort [hypericum perforatum]).

^e^ Defined as having *International Statistical Classification of Diseases and Related Health Problems, Tenth Revision *diagnosis code of hepatic impairment (K70.4, K72, K71.1) and prothrombin ratio less than 50% (ie, international normalized ratio >2.0) and the absence of an ongoing prescription of vitamin K antagonist prescription which impacts prothrombin time.

^f^ The FDA does not recommend use of nirmatrelvir-ritonavir in patients with severe hepatic and kidney impairment due to a lack of pharmacokinetic or safety data.

^g^ Glomerular fraction ratio of less than 30 mL/min/1.73 m^3^ or an *International Statistical Classification of Diseases and Related Health Problems, Tenth Revision *diagnosis code of dialysis (Z99.2, R88.0).

^h^ Patients with multiple contraindications were counted once.

## Results

Of 63 656 inpatients with COVID-19, 1131 patients (1.8%) were excluded because of missing data for sex or age. Of the 62 525 remaining patients (median [IQR] age, 52.8 [33.8-70.5] years, 31 561 [50.5%] women), 9136 (14.6%) had a medical contraindication to nirmatrelvir-ritonavir (Table 1), with higher rates in men (5568 [18.0%]) than in women (3577 [11.3%]), in older patients (5398 of 20 064 [26.9%]) than in younger ones (3738 of 42 461 [8.8%]), and in those with comorbidities (>37.0% for most comorbidities) than without comorbidities (1475 of 37 748 [3.9%]) (Table 2). Among 4861 patients who died, 2463 (50.7%) had a contraindication, with similar rates in men and women as well as older and younger patients but higher rates in patients with vs without comorbidities (Table 1 and Table 2). The most prevalent contraindications were severe kidney impairment and use of medications dependent on CYP3A for clearance.

**Table 2.** Prevalence of Possible Medical Contraindications to Nirmatrelvir-Ritonavir by *ICD-10* Main Chapters in a Sample of Patients Hospitalized With COVID-19

*ICD-10* main chapter (codes)	Full sample of hospitalized patients		Subsample of patients who died within 28 d of hospital admission	
	Patients with the condition, No.^a^	Prevalence of ≥1 possible contraindication, No. (%)	Patients with the condition, No.^a^	Prevalence of ≥1 possible contraindication, No. (%)
Certain infectious and parasitic diseases (A00-B99)	5141	2251 (43.8)	902	607 (67.3)
Neoplasms (C00-D48)	2612	999 (38.2)	657	415 (63.2)
Diseases of the blood and blood-forming organs and certain disorders involving the immune mechanism (D50-D89)	4118	1837 (44.6)	617	429 (69.5)
Endocrine, nutritional, and metabolic diseases (E00-E90)	13 592	5036 (37.1)	2355	1545 (65.6)
Mental, behavioral, and neurodevelopmental disorders (F00-F99)	5224	2058 (39.4)	1047	667 (63.7)
Diseases of the nervous system (F00-F99)	4001	1716 (42.9)	798	506 (63.4)
Diseases of the eye and adnexa (H00-H59)	824	404 (49.0)	146	111 (76.0)
Diseases of the ear and mastoid process (H60-H95)	301	128 (42.5)	34	26 (76.5)
Diseases of the circulatory system (I00-I99)	11 923	5024 (42.1)	2561	1698 (66.3)
Diseases of the respiratory system (J00-J99)	16 748	5639 (33.7)	3325	2061 (62.0)
Diseases of the digestive system (K00-K93)	3766	1565 (41.6)	589	418 (71.0)
Diseases of the skin and subcutaneous tissue (L00-L99)	1332	606 (45.5)	252	164 (65.1)
Diseases of the musculoskeletal system and connective tissue (M00-M99)	2936	1152 (39.2)	430	287 (66.7)
Diseases of the genitourinary system (N00-N99)	5628	4367 (77.6)	1473	1348 (91.5)
Pregnancy, childbirth, and the puerperium (O00-O99)	279	23 (8.24)	6	2 (33.3)
Certain conditions originating in the perinatal period (P00-P96)	85	70 (82.4)	5	1 (20.0)
Congenital malformations, deformations, and chromosomal abnormalities (Q00-Q99)	244	128 (52.5)	31	22 (71.0)
Symptoms, signs, and abnormal clinical and laboratory findings, not elsewhere classified (R00-R99)	14 099	5209 (36.9)	2659	1704 (64.1)
None of above	37 748	1475 (3.91)	1060	161 (15.2)

Abbreviation: *ICD-10*,* International Statistical Classification of Diseases and Related Health Problems, Tenth Revision*.

^a^ A patient can have multiple diseases and be counted more than once.

## Discussion

In this study, contradictions to nirmatrelvir-ritonavir were prevalent in individuals hospitalized with COVID-19, as previously suggested.^3^ These findings also alert researchers to the risk of confounding by contraindication in observational studies focused on nirmatrelvir-ritonavir, which may overestimate treatment efficacy if not excluding patients with contraindications to this treatment. In addition, some of the contraindicated medications listed here could be temporarily held in the context of using nirmatrelvir-ritonavir.^1^

Study limitations include that even if not contraindicated, treatment may not have been given to some patients due to symptom duration of longer than 5 days or limited supply, and information about vaccination, race and ethnicity, and weight was unavailable. These findings support the need to anticipate supplies for alternative approved treatments and those that are under regulatory review (eg, SARS-CoV-2 main protease inhibitors), and for continued research on less expensive treatment options for low- and middle-income countries.^4,6^
